# Mental fatigue and cognitive functioning in patients presenting with non-enhancing gliomas

**DOI:** 10.1007/s00701-025-06434-6

**Published:** 2025-03-08

**Authors:** Alice Neimantaite, Tomás Gómez Vecchio, Isabelle Rydén, Dima Harba, Asgeir S. Jakola, Anja Smits

**Affiliations:** 1 Department of Clinical Neuroscience, Institute of Neuroscience and Physiology, Sahlgrenska Academy, University of Gothenburg, Gothenburg, Sweden; 2 Department of Neurology, Sahlgrenska University Hospital, Gothenburg, Sweden; 3 Department of Neurosurgery, Sahlgrenska University Hospital, Gothenburg, Sweden

**Keywords:** Cognition, Glioma, Mental fatigue, Neuropsychological tests, Self reports

## Abstract

**Purpose:**

Patients with diffuse lower-grade gliomas (LGG) often suffer from mental fatigue. In healthy subjects, mental fatigue has a negative impact on cognitive functioning. This relation may be more complex in LGG, where tumor localization and growth rate also impact brain function. Our aim was to investigate how self- and observer-reported variables of mental fatigue and cognitive functioning were connected before tumor treatment.

**Methods:**

Consecutive patients scheduled for surgery due to presumed LGG were screened (*n* = 157). LGG was presumed if the mass was suggestive of diffuse glioma, but without significant contrast enhancement. Isocitrate dehydrogenase (IDH)-mutated WHO grade 2 or 3 gliomas (the LGG group) were analyzed separately. We included 101 patients in the entire cohort, whereas 71 patients constituted the LGG group. Patient data included: (1) self-reported assessments of mental fatigue and cognitive functioning, (2) neuropsychological test performances, and (3) clinical/demographic characteristics. Spearman's partial correlations were calculated between the variables and visualized in a correlation network.

**Results:**

Cognitive impairment was self-reported by 50% of the entire cohort and 45% of the LGG group, while observer-evaluated testing showed cognitive impairment in 40% and 34% of the cases respectively. Self-reported assessments showed no correlations (≥ 0.3 or ≤-0.3) with neuropsychological test performances. A consistent correlation was seen between self-reported mental fatigue and self-reported cognitive functioning (entire cohort: rho=-0.66, LGG group: -0.64).

**Conclusion:**

Our results highlight the complexity of evaluating symptoms of mental fatigue and cognitive functioning even prior to surgery. Self-reports and neuropsychological testing were weakly correlated, hence these should be handled complimentary.

**Supplementary Information:**

The online version contains supplementary material available at 10.1007/s00701-025-06434-6.

## Introduction

The diffuse gliomas are primary brain tumors with invasive growth, causing worsening of symptoms over time and eventually leading to death. Adult-type diffuse gliomas are graded from 2 to 4 [20]. Lower-grade gliomas (LGG) refer to diffuse gliomas of grade 2–3, i.e. isocitrate dehydrogenase (IDH)-mutated astrocytomas and oligodendrogliomas according to the most recent World Health Organization (WHO) 2021 classification [20]. Previous studies on LGG have usually included mixed cohorts of IDH-wildtype and IDH-mutated gliomas grade 2–3, due to legacy tumor classifications.

There have been significant improvements in LGG treatment in the last decade [21, 2, 15]. The prolonged survival has also increased the need to focus on symptom burden, health-related quality of life (HRQoL) and cognition [4]. Apart from seizures, patients may show cognitive deficits [31, 27], and these tend to worsen over time [4]. Similarly, patients report already at diagnosis worse HRQoL than the normal population [27]. One prominent and persistent self-reported symptom in this patient group is fatigue [33, 35, 38].

Fatigue is a broadly used term including physical, mental and other aspects [14, 18]. There is accumulating evidence that mental fatigue affects brain activity and impairs cognitive processes in healthy subjects [19, 40, 37]. In LGG, tumor localization and tumor growth rate [7, 22] have an impact on brain function, and might overshadow the relation between mental fatigue and cognitive functioning. In addition, from a clinician’s perspective, it is often a hen-and-egg situation when presenting with several problems simultaneously. Is mental fatigue mainly affecting their cognitive performance or is the cognitive impairment causing the patients’ mental fatigue? If mental fatigue is a factor causing problems in other important aspects, more research should also be directed towards reducing mental fatigue. Or vice versa, where cognitive rehabilitation potentially could reduce mental fatigue. For optimal patient counselling and rehabilitation planning, a better understanding of the relationship between mental fatigue and cognitive functioning is needed.

So far, most studies focusing on the relation between mental fatigue and cognitive functioning have used a cross-sectional design [39, 11], having the drawback of including patients at various phases of treatment. It is therefore difficult to separate tumor effect versus treatment effect [38, 6]. To study the association between mental fatigue and cognitive functioning in patients with LGG in a situation without considering treatment effects, a baseline evaluation prior to any treatment is needed. The aim of this study was to analyze the association between mental fatigue and cognitive functioning prior to surgical treatment in patients presenting with LGG (based upon Magnetic Resonance Imaging (MRI)).

## Materials and methods

### Patient inclusion

**Fig. 1:**
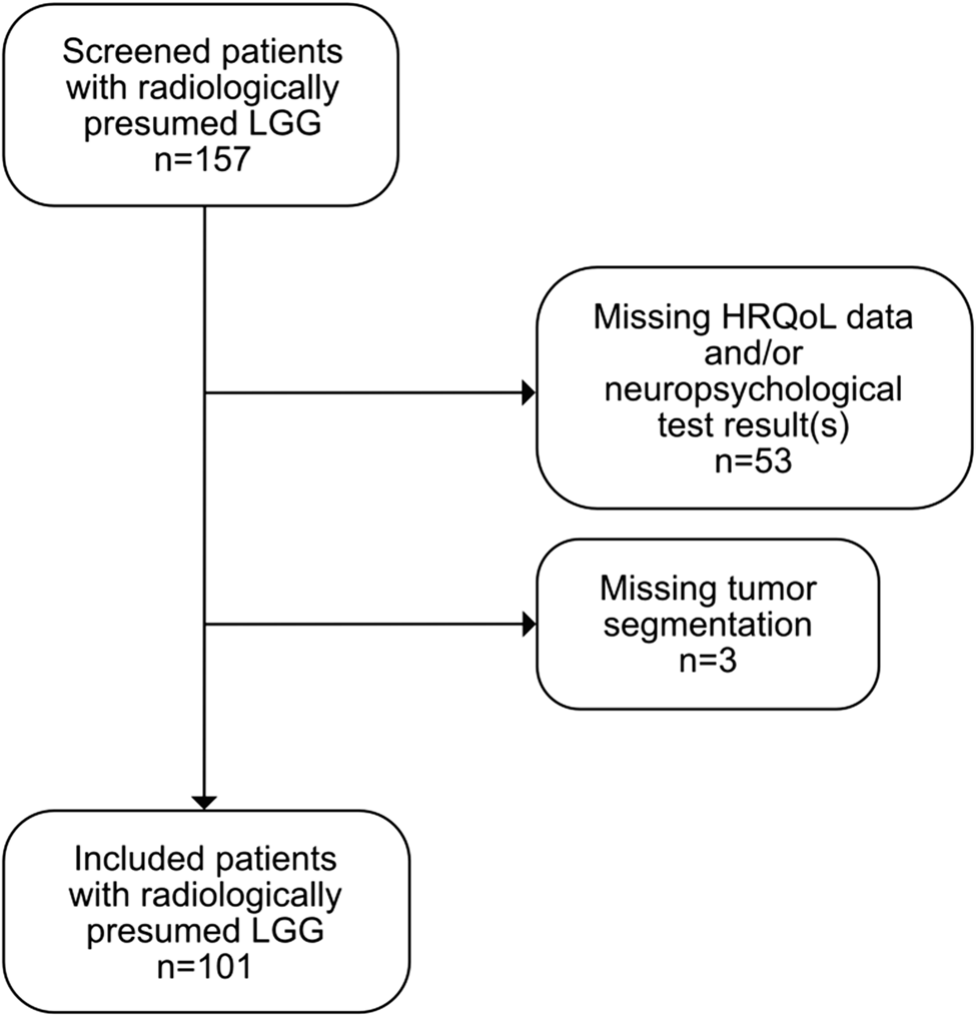
Flow-chart of patient inclusion

Patients were consecutively recruited prospectively at the Neurosurgical Department in Sahlgrenska University Hospital, Gothenburg, Sweden, from year 2017 to the first half of 2024. All patients (*n* = 157) scheduled for surgery due to a radiologically presumed LGG were invited to participate in the study. Patients were presumed to have a LGG if an intrinsic tumor mass with hyperintensity in T2-weighted images was indicating a diffuse glioma, but without significant contrast enhancement in T1-weighted images with gadolinium. Some contrast enhancement was still tolerated if other clear signs of oligodendroglioma were present, for instance calcifications [32]. Prior to operation, patients underwent clinical examination, neuropsychological testing, and were invited to fill in self-reported questionnaires. For the purpose of this study, patients with any missing data in the analyzed examinations, tests, or selected variables from the questionnaires for correlation analysis, were excluded. Consequently, we included 101 patients with presumed LGG. For details of patient inclusion and exclusion, we refer to the flow-chart (Fig. 1).

### Demographic and clinical variables

Demographic characteristics like age and gender, in addition to preoperative clinical characteristics like seizures status and Karnofsky performance status (KPS) scale [16] were registered. Tumor volumes were acquired by tumor segmentation on MRI as previously described [3]. The tumors were classified according to the WHO 2021 classification [20].

The demographic and clinical characteristics are summarized in Table 1. There were 71 patients (70.3%) with a histomolecular diagnosis of IDH-mutated WHO grade 2 or 3 glioma (the LGG group).

**Table 1:** Demographic and clinical background characteristics for patients with presumed and confirmed LGG

Variable	Presumed LGG (*N* = 101)	LGG (*N* = 71)
Age at surgery, mean (SD)	45.9 (14.0)	43.4 (13.2)
Female, n (%)	44 (43.6)	33 (46.5)
KPS^a^ <80 at admission, n (%)	8 (7.9)	4 (5.6)
Incidental finding, n (%)	5 (5.0)	4 (5.6)
Seizure debut, n (%)	71 (70.3)	50 (70.4)
AED^b^, yes, n (%)	65 (64.4)	45 (63.4)
Main tumor localization: Frontal, n (%)	45 (44.6)	36 (50.7)
Tumor hemisphere: Left, n (%)	47 (46.5)	31 (43.7)
Tumor volume, ml, median (IQR)	43.8 (53.0)	46.8 (53.4)
WHO 2021, n (%)		
IDH-mutated	78 (77.2)	71 (100.0)
Oligodendroglioma (grade 2 and 3)	35 (34.7)	35 (49.3)
Astrocytoma (grade 2 and 3)	36 (35.6)	36 (50.7)
Astrocytoma (grade 4)	7 (6.9)	-
IDH-wild type	23 (22.8)	-
Glioblastoma	13 (12.9)	-
Unclassified^c^	10 (9.9)	-

^a^ Karnofsky performance status scale, ^b^ Antiepileptic drug(s),^c^ Not able to classify with methylation analysis

### Self-reported assessments

A selection of self-reported assessments was used from the European Organization for Research and Treatment of Cancer (EORTC) Quality of Life Questionnaire C30 (QLQ-C30) [10] and the Multidimensional Fatigue Inventory (MFI-20) [30]. The following variables were selected for the analysis: EORTC QLQ-C30 Cognitive Functioning (CF), MFI-20 Mental Fatigue (MF).

EORTC QLQ-C30 Cognitive Functioning (concentration and memory), was scored 0 to 100, with higher values representing better functioning. The threshold value for clinical importance was set < 75 [12]. Mental Fatigue (concentration and mind wandering) was scored using MFI-20, on a scale 4–20, with higher values representing more severe fatigue. To our knowledge, there are no established thresholds for clinically significant symptomatology using this construct.

### Neuropsychological tests

From a larger battery of neuropsychological tests, the following were included as variables, based on their presumed association with mental fatigue: Rey Auditory Verbal Learning Test (RAVLT) [29] measuring verbal learning and memory (only delayed recall was included); Delis-Kaplan Executive Function System (D-KEFS) Phonemic Fluency (FAS) [5] measuring verbal fluency, speed and aspects of executive functioning; Trail Making Test (TMT) B [36] measuring visual attention, process speed and mental flexibility; D-KEFS Color-Word Interference Test (CWT) 4 [5] measuring interference and verbal mental flexibility (executive functioning); Wechsler Adult Intelligence Scale fourth edition (WAIS-IV) Coding (COD) [41] measuring sustained attention and mental speed; and WAIS-IV Digit Span Backwards (DIG) [41] measuring attention and working memory. For further description, see Supplementary material (Section S.1).

All neuropsychological test results were transformed into normative values t-scores. As done by others, impairment for any of the test was defined as t-score below or equal to 35, corresponding to above or equal to 1.5 standard deviations (SD) below the mean score of healthy controls [17, 23]. Per-patient impairment analysis quantified the number of patients with one or more test results showing impairment. Per-test impairment analysis was done for each test by comparison of proportion of patients with impairment compared to expected normative impairment proportion (6.68%). This analysis was done to obtain tests with significant difference in impairment proportion. For correlation analysis, t-scores were used as the neuropsychological variable values.

### Statistics

Statistics were done using IBM SPSS Statistics 28 (IBM Corp., Armonk, NY, USA). Data distribution was assessed with Q-Q plots, and Kolmogorov–Smirnov or Shapiro–Wilk normality test, depending on group size. T-test or Mann-Whitney U-test was used for continuous data group comparisons based on data normality. Fisher's exact test was used for categorical data group comparisons. Comparisons between percentages of impairments according to neuropsychological test outcomes and the expected normative value were done using Binomial test. Binomial test was calculated using Python programming language version 3.8.3 (Python Software Foundation). Tests were two sided, if not otherwise specified. Significance level was set to < 0.05. Patients with a histomolecular diagnosis showing LGG were analyzed as a separate group. Additional explorative subgroups were based on tumor localization (main lobe) and lateralization defined on MRI.

### Correlation networks

Spearman's partial correlation calculation and network visualization were implemented using Python (see Supplementary material Section S.2). For each group, Spearman's partial correlations were calculated between mental fatigue and cognitive functioning related variables, creating a partial correlation matrix. Only correlations ≥ 0.3 and ≤ −0.3 were included [1]. Accuracy analysis for correlation values was made, see Supplementary material Section S.3 for further description.

Correlation analysis results were visualized as a network of connections and correlation matrices. All network visualizations were set to nodes, arranged as a circle and with fixed node positions to facilitate comparison between networks, where nodes represented the variables of interest. Nodes were color-coded into the three categories: (1) self-reported assessments, (2) neuropsychological test performances, and (3) clinical/demographic characteristics. Connecting edges in each network represented the respective partial correlation matrix for the specified patient group. Edge thickness represented the correlation value (on middle edge position), broader edge meaning higher absolute correlation.

## Results

### Drop-out analysis, self-reported assessments and neuropsychological tests

A drop-out analysis revealed significantly more IDH-wild type tumors among the excluded patients (*p* = 0.04) (Supplementary material, Table S1). The self-reported assessments and neuropsychological test results for the included patients are reported in Table 2.

**Table 2:** The variable outcomes for patients with presumed and confirmed LGG

Variable	Presumed LGG (*N* = 101)	LGG (*N* = 71)
Self-reported assessments		
EORTC QLQ-C30^a^ – Cognitive Functioning, median (IQR)	83.3 (50.0)	83.3 (33.0)
Cognitive Functioning < 75, n (%)	50 (49.5)	32 (45.1)
MFI^b^ – Mental Fatigue, median (IQR)	11.0 (7.0)	11.0 (8.0)
Neuropsychological testing		
Patients with impairment in 1 test or more, n (%)	40 (39.6)	24 (33.8)

^a^ EORTC Quality of Life Questionnaire C-30, max score 100, ^b^ Mental Fatigue Inventory, min-max score 4–20

Per-test neuropsychological impairment rates in comparison with expected normative value, are shown in Supplementary material (Table S2, S3). The presumed LGG cohort contained a statistically significant higher proportion of patients with impairment on RAVLT, FAS and CWT (all *p* < 0.01) compared to expected normative proportion. The LGG group showed a significantly higher proportion of patients with impairment on the same tests (RAVLT *p* = 0.03, FAS *p* < 0.01, CWT *p* < 0.01) compared to expected normative proportion.

### Explorative analysis based on tumor localization and tumor laterality

#### Patients with frontal tumors compared to patients with non-frontal tumors

Demographic and clinical background characteristics, self-reported assessments and neuropsychological test performances for frontal and non-frontal tumor groups are shown in Supplementary Table S4. There was a statistically significantly different distribution of tumor types between the frontal respectively non-frontal group. In patients with frontal tumor localization, oligodendrogliomas (*p* < 0.01) were more common, while the group of non-frontal tumors included more IDH-wildtype glioblastomas (*p* = 0.03). Consequently, frontal tumors reflected more slowly growing tumors compared with the non-frontal group.

We found no statistically significant differences comparing the groups regarding self-reported assessments and neuropsychological test performances. The per-test impairments for frontal and non-frontal tumor groups separately, are supplied in the Supplementary material (Table S5, S6). A significantly higher proportion of patients with impaired results compared to expected normative proportion, irrespective of tumor localization, was seen on the CWT (frontal *p* < 0.01; non-frontal *p* = 0.03) and FAS (frontal *p* = 0.03, non-frontal *p* < 0.01). In addition, the non-frontal tumor group showed a significantly higher proportion of impaired scores on the RAVLT (*p* < 0.01) compared to expected normative proportion.

#### Patients with left hemisphere tumors compared to right hemisphere tumors

Demographic and clinical background characteristics, self-reported assessments and neuropsychological test performances comparing the group of patients with left to right hemisphere tumors are shown in the Supplementary material (Table S7). There were significantly more patients with KPS below 80 in the left hemisphere group compared with right hemisphere group (*p* = 0.02).

No statistically significant differences comparing the groups were found regarding self-reported assessments. However, significantly more patients within the left tumor localization group had ≥ 1 impaired neuropsychological test results (*p* = 0.01). Within both groups, a significantly higher proportion of patients had impaired results on CWT compared to expected normative proportion (left *p* < 0.01; right *p* = 0.03) (Supplementary material, Table S8, S9). Additionally, the left hemisphere group showed a significantly higher proportion of patients with impairment on FAS (*p* < 0.01) and RAVLT (*p* < 0.01) compared to expected normative proportion.

### Correlation networks

**Fig. 2:**
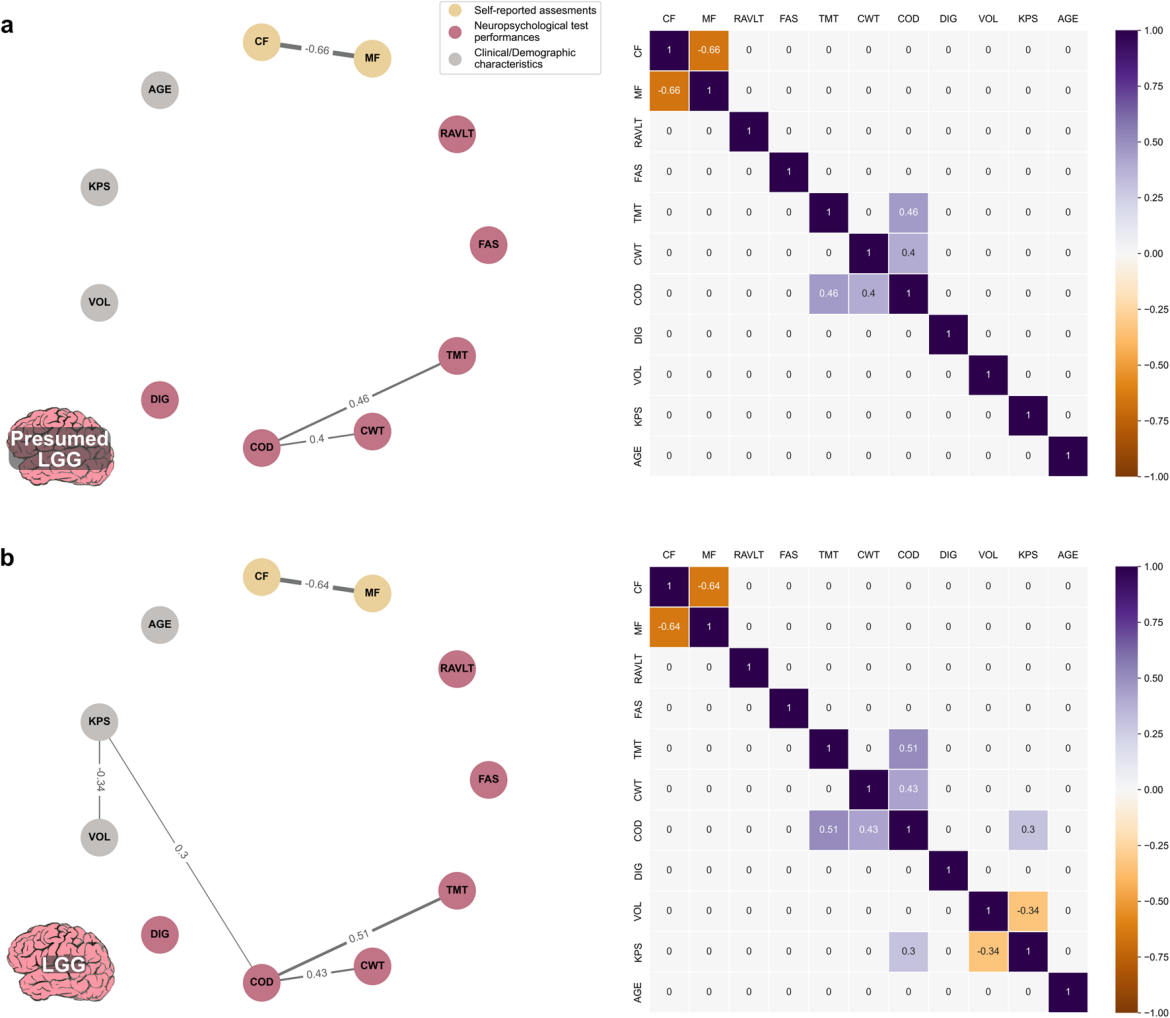
Network (left) and heatmap (right) visualizing partial correlations between the variables for a: entire patient cohort *n* = 101, b: the LGG group *n* = 71. CF = EORTC QLQ-30 Cognitive Functioning, MF = MFI-20 Mental Fatigue, RAVLT = Rey Auditory Verbal Learning Test, FAS = D-KEFS Phonemic Fluency, TMT = Trail Making Test B, CWT = D-KEFS Color-Word Interference Test, COD = WAIS-IV Coding, DIG = WAIS-IV Digit Span Backwards, VOL = Tumor volume, KPS = Karnofsky performance status

As illustrated in Fig. 2a, the cohort of patients with presumed LGG showed relatively few partial correlations (*n* = 3). There was a correlation between self-reported mental fatigue and self-reported cognitive functioning (rho=−0.66). No partial correlations between self-reported assessments and the neuropsychological test performance variables were found.

For the LGG group (Fig. 2b), similar results were seen with *n* = 5 partial correlations in total and a correlation between self-reported mental fatigue and self-reported cognitive functioning (rho=−0.64).

Correlation networks and matrices for groups of patients divided by tumor localization and tumor hemisphere, are supplied in Supplementary material Fig. S1−2. All explorative groups, based on tumor localization and tumor hemisphere, showed a correlation between self-reported mental fatigue and self-reported cognitive functioning (rho was between − 0.54 to −0.83). Frontal and non-frontal groups showed partial correlations between self-reported assessments and the neuropsychological test performances.

Accuracy analysis results are supplied in Supplementary material Section S.8. While the exact values of correlations should be read with caution, there were no large deviations from the confidence intervals, which were relatively small as well.

## Discussion

We found a consistent correlation between self-reported mental fatigue and self-reported cognitive functioning in LGG prior to operation. Patients presenting with LGG and the LGG group showed similar results regarding the correlation patterns, self-reported assessments and neuropsychological impairment. Half of the patients reported cognitive difficulties, while around one third of the patients showed impairments in neuropsychological tests. However, no correlations were seen between self-reported assessments and neuropsychological tests.

Previous cross-sectional studies in patients with gliomas have reported weak correlations between self-reported assessments and neuropsychological tests [11, 24]. One of these studies [11] found a correlation between the self-reported fatigue and self-reported cognitive functioning. Our results confirm these findings for patients with LGG in the preoperative phase. A recent study utilizing network analysis for patients with glioma, included preoperative self-reported assessment analysis, and identified fatigue as a central symptom and a possible intervention target [26]. This study is interesting but based exclusively on self-reported assessments. It is possible that our study has captured more dimensions by adding the observer-evaluated aspects, and simultaneously, our findings are narrowed to the mental part of fatigue and patients with gliomas of lower-grade.

The discrepancy between self-perceived and observed-assessed variables could be caused by several factors. For patients reporting deterioration from a premorbid high-level functioning, for example, a relative change might not be as visible in a single neuropsychological test. Another explanation could be that a decline in cognitive domains (e.g., behavioral, and social functions) may go undetected by current neuropsychological test batteries. The brain, and especially the frontal lobe, is known to process advanced cognitive functions, including executive control, language, memory, self-awareness and social behavior [34], all in which an impairment could occur and be perceived by patients. The chosen tests for this study cover several aspects of cognitive functioning, but not necessarily all functions. Also, it is still poorly understood how mental fatigue, both subjectively rated by patients and detected by neuropsychological test batteries, affects different aspects of daily life. In this context, a recent study demonstrated that cognitive tasks for choice impulsivity were markers of fatigability in patients with glioma compared to healthy controls [9].

The strengths of our study lie in the methodology, i.e. pre-surgical patient inclusion, and variables including both patient and observer perspectives. Further investigations of, if and how mental fatigue and cognitive functioning are related are highly encouraged. We also recommend analyzing larger cohorts, preferably by a quantitative tumor localization metric, instead of main tumor localization as used in this study since LGG are known to spread over several locations. Neuropsychological deficits in patients with brain tumors vary depending on tumor localization and tumor subtype [13, 8]. Fatigue, on the other hand, has not shown such a clear dependency on tumor localization [28, 25]. We observed correlations between the self-reported assessments and neuropsychological test results when dividing patients by frontal and non-frontal main tumor localization, but the groups were of relatively small sizes and further research is needed.

Although mental fatigue and cognitive problems are frequently reported by patients with LGG already prior to surgery, they do not seem easily captured by the neuropsychological assessment. Our data might suggest that rehabilitation of one patient-perceived symptom could cause significant improvement of other perceived aspects. However, this might not be reflected by improvement in clinical testing. Our findings stress the importance of evaluating this patient group by a truly multi-perspective approach and viewing observer- and self-evaluated assessments of cognition as complimentary.

## Conclusions

Self-reported mental fatigue and self-reported cognitive functioning showed a consistent correlation prior to operation in LGG. However, the self-reported outcomes did not correlate with any neuropsychological test results.

## Supplementary material

Below is the link to the electronic supplementary material.

Supplementary Material 1 (DOCX 13.0 MB)

## Data Availability

Due to the sensitive nature of the included variables in this study, the data is not available to be shared.

## Abbreviations

LGG: Lower-grade gliomas
IDH: Isocitrate dehydrogenase
WHO: World Health Organization
HRQoL: Health-related quality of life
MRI: Magnetic Resonance Imaging
KPS: Karnofsky performance status
AED: Antiepileptic drug(s)
EORTC: European Organization for Research and Treatment of Cancer
QLQ-C30: Quality of Life Questionnaire C30
MFI-20: Multidimensional Fatigue Inventory
CF: EORTC QLQ-C30 Cognitive Functioning
MF: MFI-20 Mental Fatigue
RAVLT: Rey Auditory Verbal Learning Test
FAS: Phonemic Fluency
TMT: Trail Making Test B
D-KEFS: Delis-Kaplan Executive Function System
CWT: D-KEFS Color-Word Interference Test 4
WAIS-IV: Wechsler Adult Intelligence Scale fourth edition
COD: WAIS-IV Coding
DIG: WAIS-IV Digit Span Backwards
