# Supplementary material for: Mental fatigue and cognitive functioning in patients presenting with non-enhancing gliomas
Source: Acta Neurochir (Wien). 2025 Mar 8;167(1):63. doi: 10.1007/s00701-025-06434-6 (PMC11890337; doi:10.1007/s00701-025-06434-6)
Supplement: Supplementary file 1 — Supplementary Material 1 (DOCX 13.0 MB) [file 701_2025_6434_MOESM1_ESM.docx]

**Supplementary material**

**Journal: Acta Neurochirurgica**

**Article title:** Mental fatigue and cognitive functioning in patients presenting with non-enhancing gliomas

**Authors:** Alice Neimantaite, Tomás Gómez Vecchio, Isabelle Rydén, Dima Harba, Asgeir S. Jakola, Anja Smits

**Corresponding author:** [alice.neimantaite@neuro.gu.se](mailto:alice.neimantaite@neuro.gu.se)

Department of Clinical Neuroscience, Institute of Neuroscience and Physiology, University of Gothenburg, Sahlgrenska Academy, Gothenburg, Sweden

**Section S.1. Neuropsychological testing description**

**Rey Auditory Verbal Learning Test (RAVLT):** a list of 15 words is read five times. The patient is asked to recall as many words as possible after each time. After a delay of 30 minutes the patient is asked to recall the words again, this variable aims to measure verbal memory.

**D-KEFS Verbal fluency (phonemic) (FAS):** patient is asked to generate words starting with (F, A, S) respectively, but is not allowed to use names or numbers. One minute is given for each starting letter. This test aims to measure phonemic verbal fluency, also including an executive component.

**Trail Making Test B (TMT):** the patient is asked to draw a line alternating between numbers and letters (A-1-B-2 etc) as quick as possible. The test aims to measure executive functioning (mental flexibility).

**D-KEFS Color-Word Interference Test (CWT):** the patient is presented with written color words (red, green, blue, and yellow) but the words are printed with incongruent colors. The patient is asked to name the color of the ink. However, when the words are marked by a rectangle, the patient is asked to switch to reading the words. This test measures the ability to inhibit the automatic reading process, but also to switch, which adds on another executive aspect (mental flexibility).

**WAIS IV Coding (COD):** the patient is given a list of numbers (1 to 9) corresponding to different symbols. The patient is then given a list of numbers and is asked to draw as many matching objects as possible within two minutes. The test measures processing speed and working memory.

**WAIS IV Digit Span Backwards (DIG):** a span of numbers with increasing length is read to the patient. The patient is then asked to recall the span backwards. The test aims to measure short term and working memory.

All neuropsychological test outcomes used in this study were in the form of transformed normative values, t-scores. The t-score calculation was done with normative reference values from handbook [7] for RAVLT, manual [2] for FAS and CWT, article [8] for TMT B, and manual [12] for COD and DIG.
The t-scores were corrected for gender, education and age when relevant for specific test.

**Section S.2. Python implementation for correlation analysis and network visualization**

SciPy [10] package was used for binomial test implementation. Included Python packages for correlation analysis and visualization: Pandas [6], Numpy [5], Pingouin [9], NetworkX [4], Seaborn [11], and Matplotlib [1]. Spearman partial correlations were calculated using function *partial_corr* from Pingouin.

**Section S.3. Accuracy analysis**

To evaluate the accuracy of the calculated partial correlations/edge weights, bootstrapping with replacement was applied as recommended in [3]. For each patient group, 1000 samples were drawn, and for each edge a 95% confidence interval (CI) of its mean correlation value was calculated.

In the accuracy plots, edges are ordered by the calculated partial correlation values, and for each edge, the calculated partial correlation and the 95% CI are visualized.

The following Python packages were used for accuracy analysis: NumPy, SciPy, Pingouin, Pandas and Matplotlib.

**Section S.4. Additional results: Drop-out analysis**

**Table S1.** Drop-out analysis between included patients and excluded patients due to missing data.

| **Variable** | **Included patients (N=101)** | **Excluded patients (N=56)** | **p-value**^a^ |
| --- | --- | --- | --- |
| Age at surgery, mean (SD) | 45.9 (14.0) | 49.8 (14.5) | 0.11 |
| Female, n (%) | 44 (43.6) | 19 (33.9) | 0.31 |
| KPS^b^ <80 at admission, n (%) | 8 (7.9) | 5 (8.9) | 1.00 |
| Incidental finding, n (%) | 5 (5.0) | 6 (10.7) | 0.20 |
| Seizure debut, n (%) | 71 (70.3) | 35 (62.5) | 0.38 |
| AED^c^, yes, n (%) | 65 (64.4) | 35 (62.5) | 0.86 |
| Main tumor localization: Frontal, n (%) | 45 (44.6) | 24 (42.9) | 0.87 |
| Tumor hemisphere: Left, n (%) | 47 (46.5) | 25 (44.6) | 0.87 |
| WHO 2021, n (%) |  |  |  |
| IDH-mutated | 78 (77.2) | 34 (60.7) | 0.04 |
| Oligodendroglioma (grade 2 and 3) | 35 (34.7) | 17 (30.4) | 0.60 |
| Astrocytoma (grade 2 and 3) | 36 (35.6) | 16 (28.6) | 0.38 |
| Astrocytoma (grade 4) | 7 (6.9) | 1 (1.8) | 0.26 |
| IDH-wild type | 23 (22.8) | 22 (39.3) | 0.04 |
| Glioblastoma | 13 (12.9) | 9 (16.1) | 0.63 |
| Unclassified^d^ | 10 (9.9) | 13 (23.2) | 0.03 |

^a^ Independent t-test, Fisher’s exact test, ^b^ Karnofsky performance status scale, ^c^ Antiepileptic drug(s),^d^ Not able to classify with methylation analysis.

**Section S.5. Additional results: Patients with presumed and confirmed LGG**

**Table S2.** Per-test neuropsychological impairment rate compared with expected normative percentage of 6.68% for all patients with presumed LGG (n=101).

| **Neuropsychological test** | **Impairment rate in patients, n (%)** | **p-value^a^** |
| --- | --- | --- |
| Rey Auditory Verbal Learning Test | 19 (18.8) | <.01 |
| Phonemic Fluency | 17 (16.8) | <.01 |
| Trail Making Test B | 10 (9.9) | 0.23 |
| Color-Word Interference Test | 17 (16.8) | <.01 |
| Coding | 7 (6.9) | 0.84 |
| Digit Span Backwards | 4 (4.0) | 0.42 |

^a^Binomial test.

**Table S3.** Per-test neuropsychological impairment rate compared with expected normative percentage of 6.68% for the LGG group (n=71).

| **Neuropsychological test** | **Impairment rate in patients, n (%)** | **p-value^a^** |
| --- | --- | --- |
| Rey Auditory Verbal Learning Test | 10 (14.1) | 0.03 |
| Phonemic Fluency | 12 (16.9) | <.01 |
| Trail Making Test B | 7 (9.9) | 0.33 |
| Color-Word Interference Test | 11 (15.5) | <.01 |
| Coding | 3 (4.2) | 0.63 |
| Digit Span Backwards | 4 (5.6) | 1.0 |

^a^Binomial test.

**Section S.6. Additional results: Patients with frontal compared with non-frontal tumors**

Non-frontal tumors included main localization in temporal lobe (n=27), insular lobe (n=15), parietal lobe (n=11), occipital lobe (n=1) and basal ganglia/thalamus (n=2).

**Table S4.** Demographic and clinical background characteristics, and variable outcomes in patients grouped by main tumor localization.

| **Variable** | **Frontal tumors (N=45)** | **Non-frontal  tumors (N=56)** | **p-value**^a^ |
| --- | --- | --- | --- |
| Age at surgery, mean (SD) | 44.0 (13.9) | 47.5 (14.0) | 0.22 |
| Female, n (%) | 24 (53.3) | 20 (35.7) | 0.11 |
| KPS^b^ <80 at admission, n (%) | 4 (8.9) | 4 (7.1) | 1.00 |
| Incidental finding, n (%) | 1 (2.2) | 4 (7.1) | 0.38 |
| Seizure debut, n (%) | 30 (66.7) | 41 (73.2) | 0.52 |
| AED^c^, yes, n (%) | 27 (60.0) | 38 (67.9) | 0.53 |
| Tumor hemisphere: Left, n (%) | 20 (44.4) | 27 (48.2) | 0.84 |
| Tumor volume, ml, median (IQR) | 44.9 (66.9) | 43.3 (44.6) | 0.99 |
| WHO 2021, n (%) |  |  |  |
| IDH-mutated | 39 (86.7) | 39 (69.6) | 0.06 |
| Oligodendroglioma (grade 2 and 3) | 23 (51.1) | 12 (21.4) | <.01 |
| Astrocytoma (grade 2 and 3) | 13 (28.9) | 23 (41.1) | 0.22 |
| Astrocytoma (grade 4) | 3 (6.7) | 4 (7.1) | 1.00 |
| IDH-wild type glioma | 6 (13.3) | 17 (30.4) | 0.06 |
| Glioblastoma | 2 (4.4) | 11 (19.6) | 0.03 |
| Unclassified^d^ | 4 (8.9) | 6 (10.7) | 1.00 |
| Self-reported assessments |  |  |  |
| EORTC QLQ-C30^e^ – Cognitive Functioning, median (IQR) | 83.3 (50) | 75.2 (46) | 0.91 |
| Cognitive Functioning < 75, n (%) | 22 (48.9) | 28 (50.0) | 1.00 |
| MFI^f^ – Mental Fatigue, median (IQR) | 11.0 (6.0) | 11.5 (7.0) | 0.85 |
| Neuropsychological testing |  |  |  |
| Patients with impairment in 1 test or more, n (%) | 15 (33.3) | 25 (44.6) | 0.31 |

^a^ Independent t-test, Mann-Whitney U test, Fisher’s exact test, ^b^ Karnofsky performance status scale, ^c^ Antiepileptic drug(s), ^d^ Not able to classify with methylation analysis, ^e^ EORTC Quality of Life Questionnaire C-30, max score 100, ^f^ Mental Fatigue Inventory, min-max score 4-20.

**Table S5.** Per-test neuropsychological impairment rate in patients with frontal tumors (n=45) compared with expected normative percentage of 6.68%.

| **Neuropsychological test** | **Impairment rate in patients, n (%)** | **p-value^a^** |
| --- | --- | --- |
| Rey Auditory Verbal Learning Test | 3 (6.7) | 1.00 |
| Phonemic Fluency | 7 (15.6) | 0.03 |
| Trail Making Test B | 5 (11.1) | 0.22 |
| Color-Word Interference Test | 9 (20.0) | <.01 |
| Coding | 3 (6.7) | 1.00 |
| Digit Span Backwards | 3 (6.7) | 1.00 |

^a^Binomial test.

**Table S6.** Per-test neuropsychological impairment rate in patients with non-frontal tumors (n=56) compared with expected normative percentage of 6.68%.

| **Neuropsychological test** | **Impairment rate in patients, n (%)** | **p-value^a^** |
| --- | --- | --- |
| Rey Auditory Verbal Learning Test | 16 (28.6) | <.01 |
| Phonemic Fluency | 10 (17.9) | <.01 |
| Trail Making Test B | 5 (8.9) | 0.42 |
| Color-Word Interference Test | 8 (14.3) | 0.03 |
| Coding | 4 (7.1) | 0.79 |
| Digit Span Backwards | 1 (1.8) | 0.18 |

^a^Binomial test.


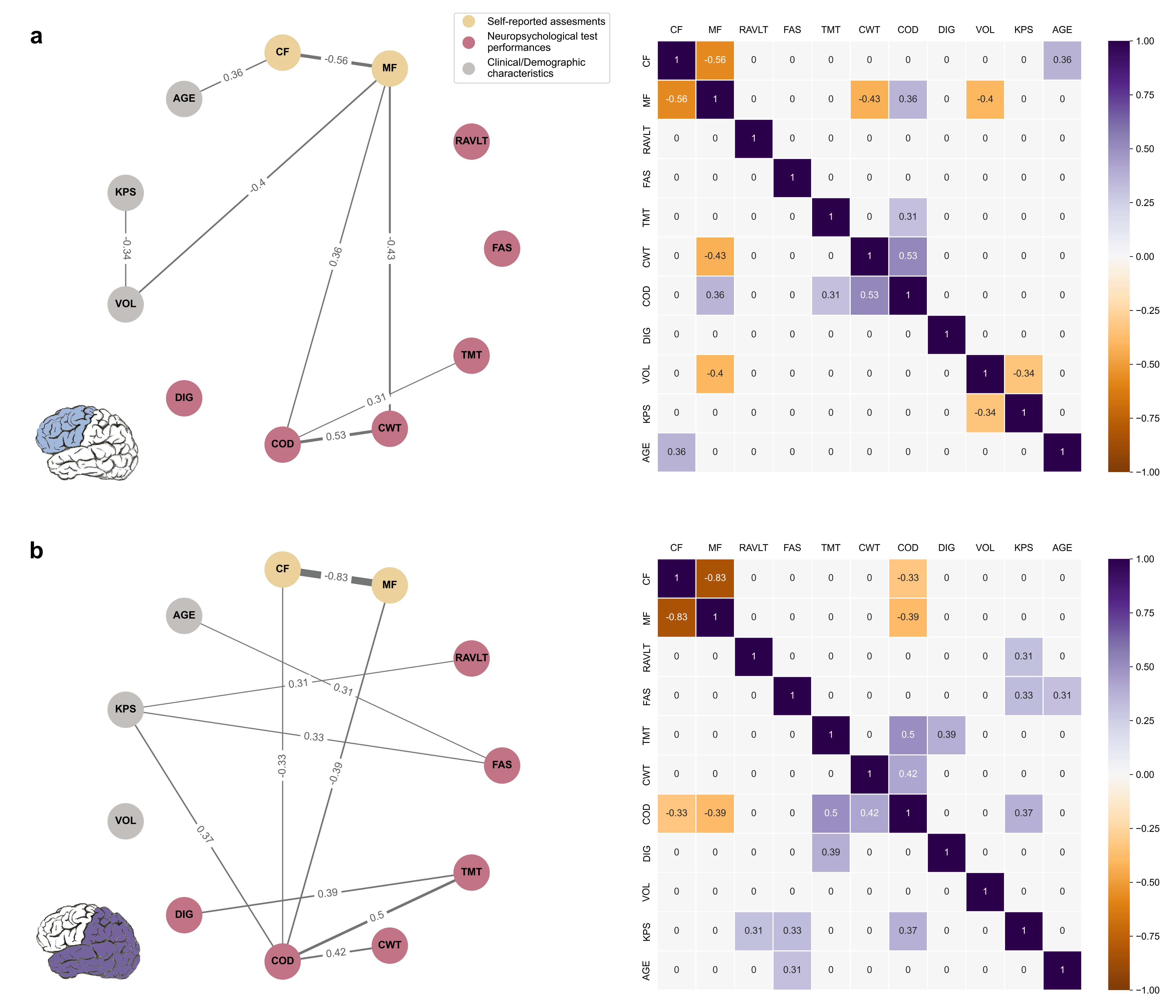


**Fig. S1** Network (left) and heatmap (right) visualizing partial correlations between the variables for a: patients with frontal tumors n=45, b: patients with non-frontal tumors n=56. CF = EORTC QLQ-30 Cognitive Functioning, MF = MFI-20 Mental Fatigue, RAVLT = Rey Auditory Verbal Learning Test, FAS = D-KEFS Phonemic Fluency, TMT = Trail Making Test B, CWT = D-KEFS Color-Word Interference Test, COD = WAIS-IV Coding, DIG = WAIS-IV Digit Span Backwards, VOL = Tumor volume, KPS = Karnofsky performance status

**Section S.7. Additional results: Patients with left compared with right hemisphere tumors**

**Table S7.** Demographic and clinical background characteristics, and variable outcomes in patients grouped by tumor hemisphere.

| **Variable** | **Left side tumors (N=47)** | **Right side  tumors (N=54)** | **p-value**^a^ |
| --- | --- | --- | --- |
| Age at surgery, mean (SD) | 46.7 (13.1) | 45.3 (14.9) | 0.62 |
| Female, n (%) | 22 (46.8) | 22 (40.7) | 0.55 |
| KPS^b^ <80 at admission, n (%) | 7 (14.9) | 1 (1.9) | 0.02 |
| Incidental finding, n (%) | 3 (6.4) | 2 (3.7) | 0.66 |
| Seizure debut, n (%) | 32 (68.1) | 39 (72.2) | 0.67 |
| AED^c^, yes, n (%) | 32 (68.1) | 33 (61.1) | 0.54 |
| Main tumor localization: Frontal, n (%) | 20 (42.6) | 25 (46.3) | 0.84 |
| Tumor volume, ml, median (IQR) | 44.1 (74.1) | 39.4 (40.7) | 0.75 |
| WHO 2021, n (%) |  |  |  |
| IDH-mutated | 36 (76.6) | 42 (77.8) | 1.00 |
| Oligodendroglioma (grade 2 and 3) | 14 (29.8) | 21 (38.9) | 0.40 |
| Astrocytoma (grade 2 and 3) | 17 (36.2) | 19 (35.2) | 1.00 |
| Astrocytoma (grade 4) | 5 (10.6) | 2 (3.7) | 0.25 |
| IDH-wild type glioma | 11 (23.4) | 12 (22.2) | 1.00 |
| Glioblastoma | 6 (12.8) | 7 (13.0) | 1.00 |
| Unclassified^d^ | 5 (10.6) | 5 (9.3) | 1.00 |
| Self-reported assessments |  |  |  |
| EORTC QLQ-C30^e^ – Cognitive Functioning, median (IQR) | 67.0 (50.0) | 83.3 (38.0) | 0.54 |
| Cognitive Functioning < 75, n (%) | 25 (53.2) | 25 (46.3) | 0.55 |
| MFI^f^ – Mental Fatigue, median (IQR) | 11.0 (7.0) | 11.5 (7.0) | 0.96 |
| Neuropsychological testing |  |  |  |
| Patients with impairment in 1 test or more, n (%) | 25 (53.2) | 15 (27.8) | 0.01 |

^a^ Independent t-test, Mann-Whitney U test, Fisher’s exact test, ^b^ Karnofsky performance status scale, ^c^ Antiepileptic drug(s), ^d^ Not able to classify with methylation analysis, ^e^ EORTC Quality of Life Questionnaire C-30, max score 100, ^f^ Mental Fatigue Inventory, min-max score 4-20.

**Table S8.** Per-test neuropsychological impairment rate in patients with left hemisphere tumors (n=47) compared with expected normative percentage of 6.68%.

| **Neuropsychological test** | **Impairment rate in patients, n (%)** | **p-value^a^** |
| --- | --- | --- |
| Rey Auditory Verbal Learning Test | 13 (27.7) | <.01 |
| Phonemic Fluency | 13 (27.7) | <.01 |
| Trail Making Test B | 5 (10.6) | 0.24 |
| Color-Word Interference Test | 9 (19.1) | <.01 |
| Coding | 2 (4.3) | 0.77 |
| Digit Span Backwards | 3 (6.4) | 1.00 |

^a^Binomial test.

**Table S9.** Per-test neuropsychological impairment rate in patients with right hemisphere tumors (n=54) compared with expected normative percentage of 6.68%.

| **Neuropsychological test** | **Impairment rate in patients, n (%)** | **p-value^a^** |
| --- | --- | --- |
| Rey Auditory Verbal Learning Test | 6 (11.1) | 0.17 |
| Phonemic Fluency | 4 (7.4) | 0.78 |
| Trail Making Test B | 5 (9.3) | 0.41 |
| Color-Word Interference Test | 8 (14.8) | 0.03 |
| Coding | 5 (9.3) | 0.41 |
| Digit Span Backwards | 1 (1.9) | 0.27 |

^a^Binomial test.


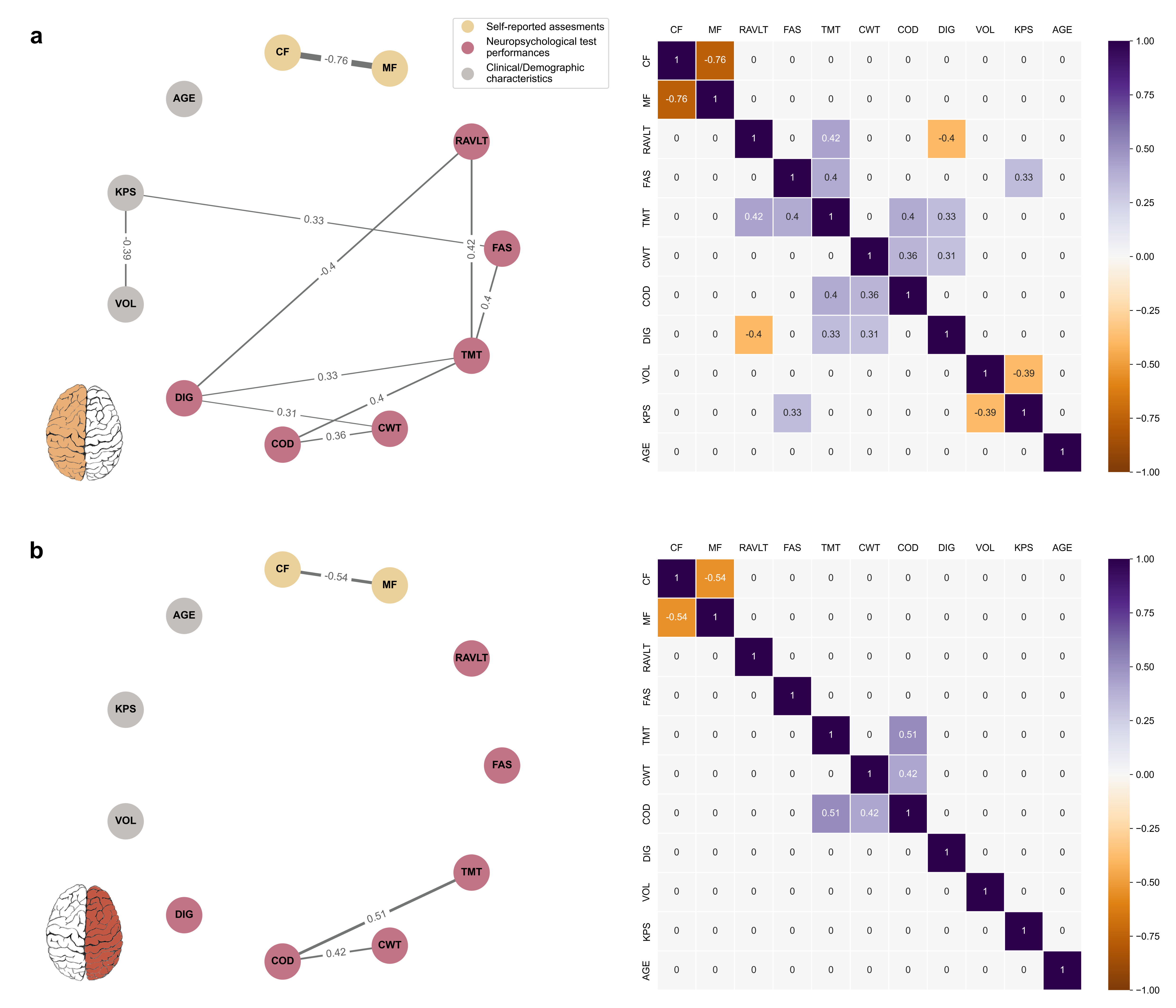


**Fig. S2** Network (left) and heatmap (right) visualizing partial correlations between the variables for a: patients with left hemisphere tumors n=47, b: patients with right hemisphere tumors n=54. CF = EORTC QLQ-30 Cognitive Functioning, MF = MFI-20 Mental Fatigue, RAVLT = Rey Auditory Verbal Learning Test, FAS = D-KEFS Phonemic Fluency, TMT = Trail Making Test B, CWT = D-KEFS Color-Word Interference Test, COD = WAIS-IV Coding, DIG = WAIS-IV Digit Span Backwards, VOL = Tumor volume, KPS = Karnofsky performance status

**Section S.8. Additional results: Accuracy analysis**

Accuracy plots for each patient group are visualized below.

There appears to be slight uncertainty in the exact values of the calculated partial correlations. However, the CIs are relatively small and the calculated partial correlation values are close to the CIs, especially regarding the included relations after thresholding (in green), indicating accuracy of the included relations.

**
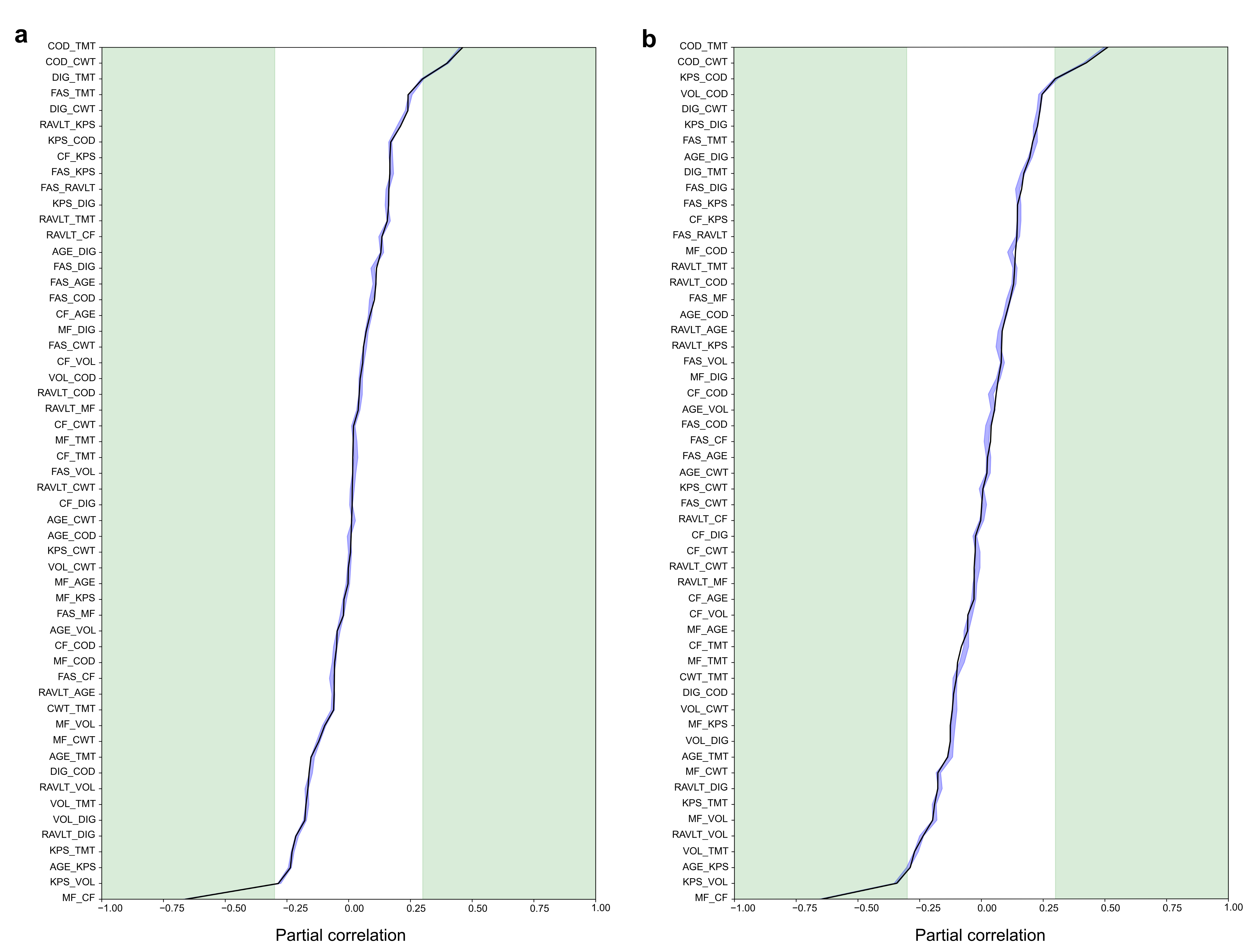
**

**Fig. S3** a: Accuracy analysis of partial correlations for a) all included patients (presumed LGG) (n=101) b) patients with LGG (n=71). The black line shows partial correlations calculated on the specified group. The blue interval shows the 95% CI for partial correlations by bootstrap samples of the specified group. The green intervals show included correlation interval

**
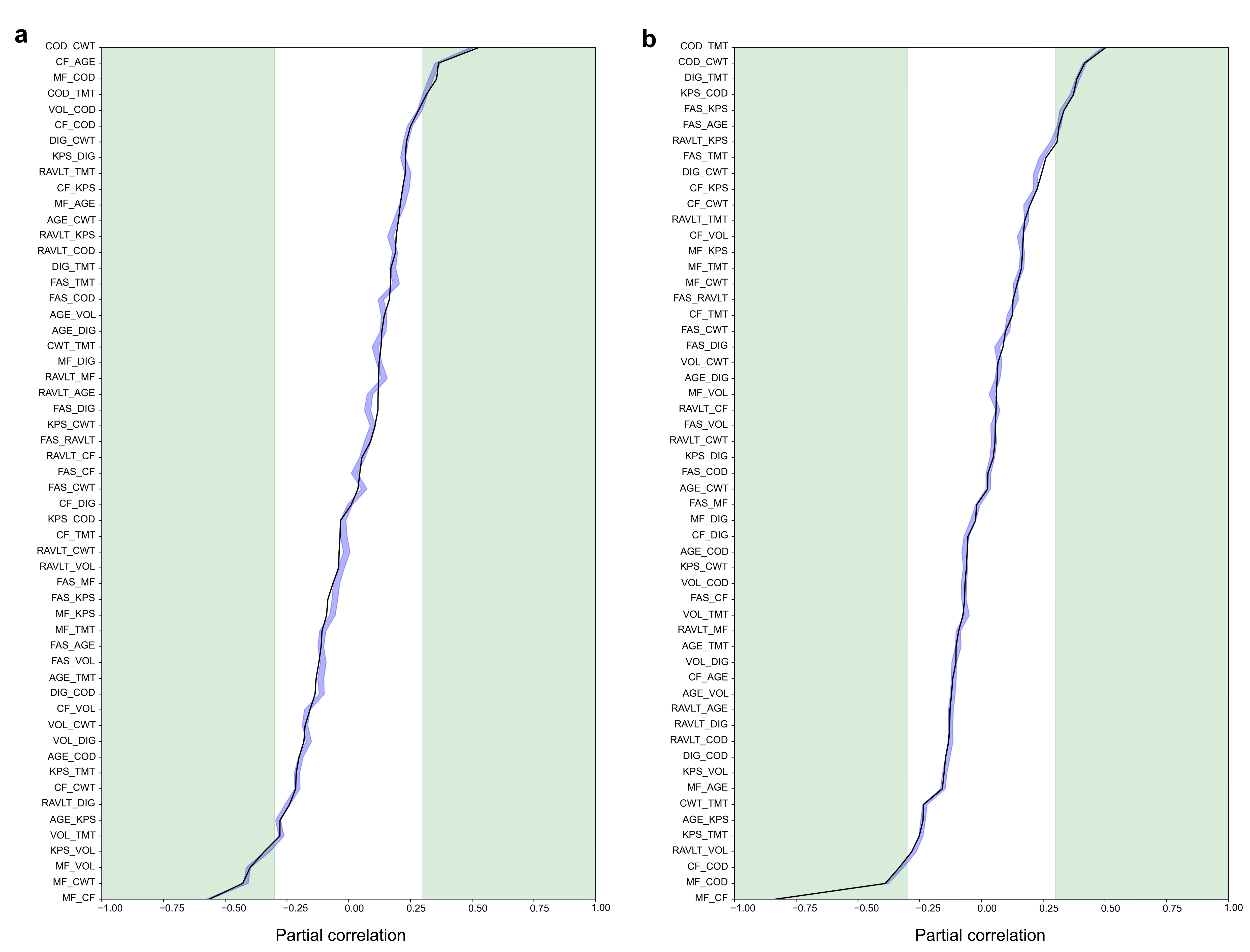
**

**Fig. S4** a: Accuracy analysis of partial correlations for a: patients with frontal tumors n=45, and b: patients with non-frontal tumors n=56. The black line shows partial correlations calculated on the specified group. The blue interval shows the 95% CI for partial correlations by bootstrap samples of the specified group. The green intervals show included correlation interval

**
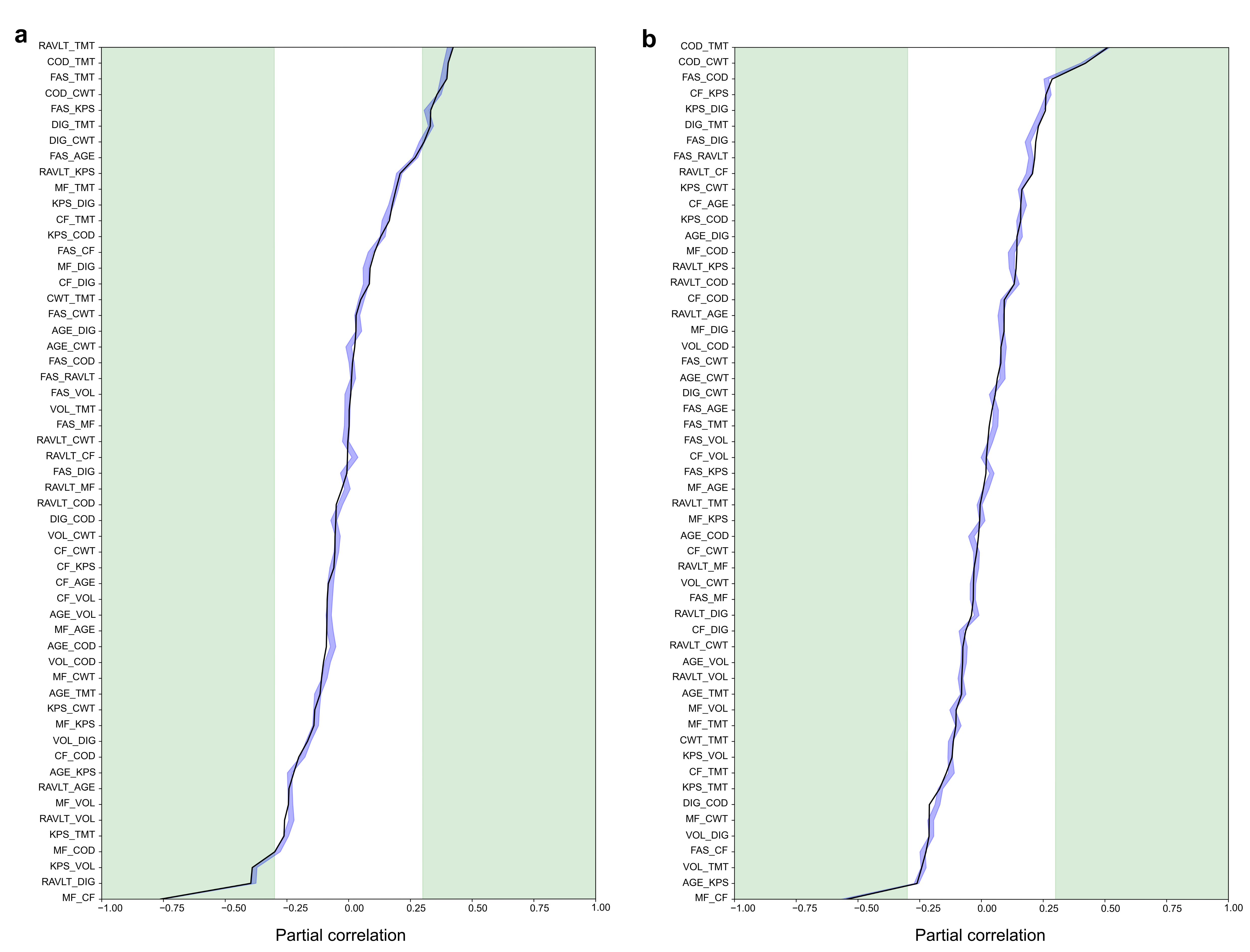
**

**Fig. S5** a: Accuracy analysis of partial correlations for a: patients with left hemisphere tumors n=47, and b: patients with right hemisphere tumors n=54. The black line shows partial correlations calculated on the specified group. The blue interval shows the 95% CI for partial correlations by bootstrap samples of the specified group. The green intervals show included correlation interval

**References**

1. Barrett P, Hunter J, Miller JT, Hsu J-C, Greenfield P matplotlib--A Portable Python Plotting Package. In: Astronomical data analysis software and systems XIV, 2005. p 91

2. Delis DC, Kaplan E, Kramer JH (2001) Delis - Kaplan executive function system Examiner’s Manual. The Psychological Corporation, San Antonio, TX

3. Epskamp S, Borsboom D, Fried EI (2018) Estimating psychological networks and their accuracy: A tutorial paper. Behavior research methods 50:195-212

4. Hagberg A, Swart P, S Chult D (2008) Exploring network structure, dynamics, and function using NetworkX. Los Alamos National Lab.(LANL), Los Alamos, NM (United States),

5. Harris CR, Millman KJ, Van Der Walt SJ, Gommers R, Virtanen P, Cournapeau D, Wieser E, Taylor J, Berg S, Smith NJ (2020) Array programming with NumPy. Nature 585:357-362

6. McKinney W (2011) pandas: a foundational Python library for data analysis and statistics. Python for high performance and scientific computing 14:1-9

7. Schmidt M (1996) Rey auditory verbal learning test: A handbook, vol 17. Western Psychological Services Los Angeles, CA,

8. Tombaugh TN (2004) Trail Making Test A and B: normative data stratified by age and education. Archives of clinical neuropsychology 19:203-214

9. Vallat R (2018) Pingouin: statistics in Python. J Open Source Softw 3:1026

10. Virtanen P, Gommers R, Oliphant TE, Haberland M, Reddy T, Cournapeau D, Burovski E, Peterson P, Weckesser W, Bright J (2020) SciPy 1.0: fundamental algorithms for scientific computing in Python. Nature methods 17:261-272

11. Waskom ML (2021) Seaborn: statistical data visualization. Journal of Open Source Software 6:3021

12. Wechsler D (2011) Wechsler Adult Intelligence Scale - Fourth Edition Manual Del 1, Svensk version. Pearson, Stockholm
